# A Simple Queuing Model for Molecular Communications Receivers

**DOI:** 10.3390/s21227664

**Published:** 2021-11-18

**Authors:** Mauro Femminella, Gianluca Reali

**Affiliations:** 1 Department of Engineering, University of Perugia, Via G. Duranti 93, 06125 Perugia, Italy; mauro.femminella@unipg.it; 2 Consorzio Nazionale Interuniversitario per le Telecomunicazioni (CNIT), 43124 Parma, Italy

**Keywords:** molecular communications, diffusion, markovian model, scalable simulation

## Abstract

The complexity of molecular communications system, involving a massive number of interacting entities, makes scalability a fundamental property of simulators and modeling tools. A typical scenario is that of targeted drug delivery systems, which makes use of biological nanomachines close to a biological target, able to release molecules in the diseased area. In this paper, we propose a simple but reliable receiver model for diffusion-based molecular communication systems tackling the time needed for analyzing such a system. The proposed model consists of using an equivalent markovian queuing model, which reproduces the aggregate behavior of thousands of receptors spread over the receiver surface. It takes into account not only the fact that the absorption of molecules can occur only through receptors, but also that absorption is not an instantaneous process and may require a significant time during which the receptor is not available to bind to other molecules. Our results, expressed in terms of number of absorbed molecules and average number of busy receptors, demonstrate that the proposed approach is in good agreement with results obtained through particle-based simulations of a large number of receptors, although the time taken for obtaining the results with the proposed model is an order of magnitudes lower than the simulation time. We believe that this model can be the precursor of novel class of models based on similar principles that allow realizing reliable simulations of much larger systems.

## 1. Introduction and Background

Molecular communication is a novel paradigm allowing information exchange by means of molecules between biological nanomachines over short ranges [1,2]. Biological, chemical, and physical processes are involved in the establishment of molecular communications, which makes the resulting context quite challenging. Molecular communications is gaining momentum due to the possible innovative applications that can be realized in many fields, with a special focus on the medical one [3]. Among them, targeted drug delivery is currently regarded as one of the most promising. The various relevant proposals are illustrated in [4], which illustrates a simple model to address complexity in drug delivery leveraging molecular communications.

In the simplest scenario for molecular communications, a transmitter nanomachine (TX) emits molecules, which propagate and eventually are received by a receiver nanomachine (RX), which in turn decodes the relevant information by processing them. The interested reader can refer to the work in [5] for a review of end-to-end communication schemes. The information can be encoded on the concentration of the emitted particles, on the frequency of their emission, and/or on the type of released molecules. Depending on the target of the communications, it can be realized by emitting a number of symbols composed by a burst of molecules, each one carrying its own information content as in classic digital communications, or in a sustained flow of molecules, as in the case of drug delivery. The information molecules can interact with the receiver node in several ways, which may induce a variation of their concentration around the receiver. The simplest reception model assumes an ideal passive (known also as *transparent*) receiver, which is permeable to the hitting molecules and is capable only to count the number of the molecules inside its volume, without any physical or chemical interaction with them. This means also that the hitting molecules will eventually contribute to the received signal more than once in different symbol intervals, until they are inside the volume of the transparent RX. To go further, a more realistic molecular communication system is the so-called absorbing receiver, which removes each molecule from the environment after the absorption so that it contributes only once to the received signal. In the simplest form, the absorption takes place through the receiver surface, which may happen for very small molecules, able to pass through the the external membrane of the receiver node. In a more sophisticated and realistic model, especially for large or polar signaling molecules that cannot passively diffuse through the membrane of cells, they can be absorbed only through the receptors that cover the external membrane of the receiver node. This mechanism can be described by a form of chemical reaction, as the reception process consists of a chemical reaction between the signal molecules (ligand) and compliant receptors present on the RX surface. These receptors are able to react only with specific types of information molecules, thus each cell/nanomachine will have a number of different receptors to receive different signals. For such reactive receivers, the sensing area is not the whole cell surface, but it is just that part of the receiver surface that is covered by receptors, and the number of activated receptors form the received signal. In more detail, these receptors may absorb or bind with the information molecules. In the first case, the ligand–receptor chemical complex formed by the ligand and the receptor is internalized by the nanomachine and follows a process known as trafficking [6]. In the latter case, it may happen that some molecules desorb from their receptors (reversible reaction) and may contribute several times to the received signal (see also in [7]). In both cases, the received signaling molecules may trigger inside the receiving cell/nanomachine the production of secondary molecules via the so-called signaling pathways. These secondary molecules can later be used for detection or decoding of the information, or to trigger additional complex reactions. In nature, cells present a number of signaling pathways, each one responsible for relaying a particular type of measurement taken in the extracellular space to the organelles in the cytosol, which ultimately causes a response by the cell. For instance, very complex interactions are those triggered by the absorption of the complex formed by the spike protein of SARS-CoV-2 with the ACE2 receptor of human cells (see in [8] and the references therein).

When a reactive receiver is considered, it is necessary to bear in mind the concept of receiver congestion, known as saturation in biology, which is of great importance in drug delivery systems [9]. Saturation is due to the reaction time of ligands with receptors, especially when the average time during which a receptor is busy becomes significant. From a macroscopic viewpoint, saturation is modeled by means of the so-called reaction rates [10]. A more detailed, microscopic model is proposed in [11]. However, as already pointed out in [9], this model, which uses a receptor pool, does not take into account the fact that each receptor operates in isolation, and thus cannot account for a correct estimation of failed molecule assimilation attempts (rejections). This problem is addressed in [9], which proposes to address each receptor in isolation, to correctly evaluate assimilation and rejection rates upon a given transmission rate. However, that model depends on the displacement of receptors on the receiver surface, which, in general, is not known in advance. In this paper, we show that approximating all receptors as they were concentrated in a single point provides a very good agreement with simulation results, even if each of them is modeled singularly by a pure loss queuing system.

Thus, in this paper, we propose a RX model for diffusion-based molecular communications, which could allow designing scalable simulation tools for molecular communications. In the literature, we can find other initiatives aiming at designing scalable simulators, addressing the problem in a different way. For example, in [12] the authors partition the overall simulation volume of a molecular communications system through both flow and diffusion components in adjacent homogeneous mesoscopic subvolumes. The rates of transition of particles from one subvolume to another can be integrated in stochastic simulations of reaction–diffusion systems that follow a mesoscopic approach, i.e., application of a spatial stochastic simulation algorithm. This means that simulation of particle movement is more coarse when it happens far from TX or RX nodes and becomes accurate close to them. Another approach consists of using space partitions and GPUs to detect collision among different entities (ligands, RX, TX, receptors on RX surface) to decrease the simulation time [13]. Both approaches do not eliminate the simulation of particle movements, but make them more approximate and faster. Differently, the rationale of our work consists of avoiding the individual simulation of the movement of thousands of particles, which is replaced by the use of a model that describes the dynamic interaction between a subset of particles and their compliant receptors. In more detail, we consider a molecular communication system where a nearly continuous flow of molecules is released from the TX. Such molecules, which are aimed to be received by the RX, may happen to be grouped in consecutive bursts, in order to obtain a desired effective average rate of molecules at a specific target. A number of receptors is present over the RX surface. Each of them is specialized to match a ligand type and can make a bond at a time with a compliant ligand. As in the considered scenario the space around the RX could become full of signal molecules, congestion may arise. It may result in the inability of busy receptors to build additional bonds with colliding ligand molecules. We model this interaction by means of the queuing theory. In particular, we model each receptor as an M/M/1/1 queue in order to take into account saturation phenomena.

This paper expands the preliminary results presented as a poster in [14]. In particular, we present a more detailed and complete model, along with the relevant simulation results. We compare the results obtained by using two sets of numerical results, using the proposed queuing model and a particle based simulator, respectively. The results are expressed in terms of busy receptors and average number of absorbed molecules.

In Section 2, we provide the system model, whereas, in Section 3, we assess the proposed model by means of simulation and reports some relevant consideration. Finally, in Section 4, we report our concluding remarks.

## 2. System Model and Performance Analysis

**Figure 1:**
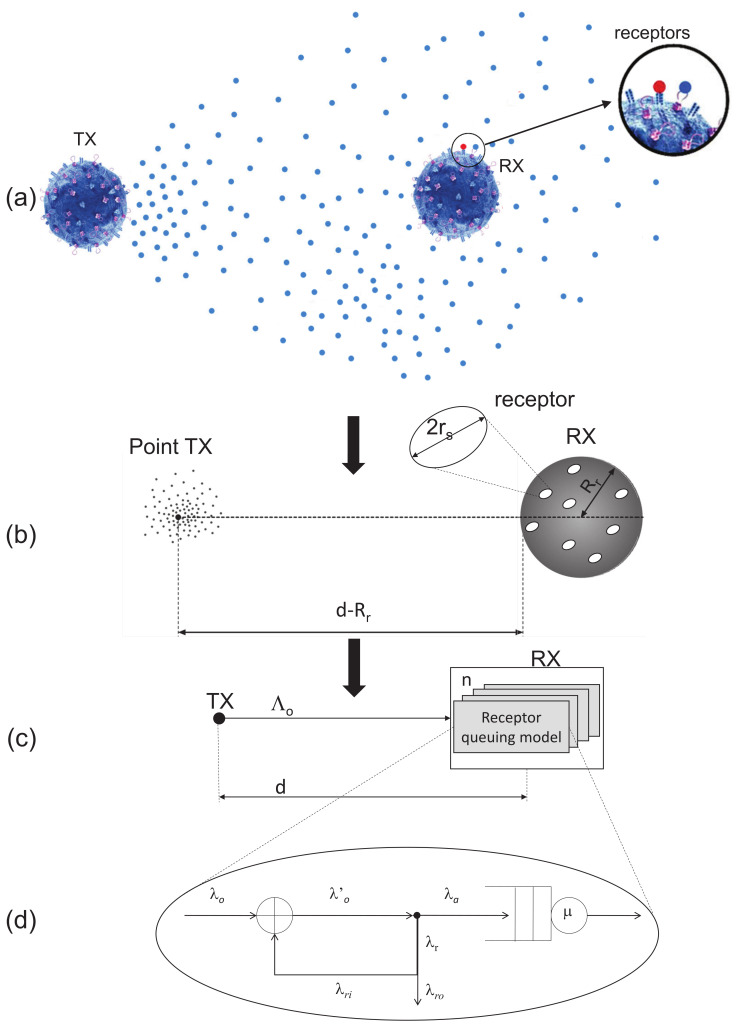
Overall system model: (**a**) emitting and receiving nanomachines with surface receptors, (**b**) equivalent molecular communications model with receptors of radius $r_{s}$, (**c**) queuing model of the RX with all *n* receptors, and (**d**) queuing model for each receptor.

The proposed system model is sketched in Figure 1. This figure illustrates all the logical steps staying behind the definition of our model. First, Figure 1a illustrates the physical model in which a transmitting nanomachine, the TX, releases a number of ligands in the surrounding 3D environment. Through the propagation by diffusion, these ligands can reach the receiving nanomachine, the RX node, which is equipped with a number of different receptors. Only compliant receptors can bind with the released ligands. This behavior is typically modeled in molecular communications literature as a point TX, releasing particles that diffuse through the surrounding 3D environment, and a spherical RX, whose surface is covered by circular receptors of radius $r_{s}$, as illustrated in Figure 1b (see, e.g., in [15]). We further model this system as a set of queues, one for each receptor. We make the simplified assumption that all receptors are *virtually* located in the same point, as we prefer avoiding to make specific assumptions on the position of receptors on the RX surface [16] (Figure 1c). Finally, Figure 1d represents the single receptor service system through the well-known M/M/1/1 queue. The use of queuing models for molecular communication systems is not new, as shown in [9,11,17]. The original aspect in this paper is that it is used to develop a simplified model to be used in large-scale simulations, that represent a challenging task when such systems are analyzed. We model a receptor as a server with an exponential service time (trafficking time) with mean to 1/μ.

In order to model a continuous transmission of molecules from the TX, which may represent a drug delivery process, we assume that the TX node transmits a pulse of *Q* molecules every Δt seconds, starting from t=0. These molecules propagate in the communication environment departing from the TX according to the law of diffusion [18]. The propagation environment is characterized by the diffusion coefficient, given by $D=\frac{k_{B}T}{6\pi \eta r_{m}}$, where $k_{B}$ is the Boltzmann constant, *T* is the temperature expressed in kelvins, η is the viscosity of the medium, and $r_{m}$ is the radius of the considered molecules. If we use the simplified receiver model consisting of a receiver cell with absorbing receptors, the resulting system would be linear, and the resulting effect would consist of the superposition of the solution for each pulse of molecules [15,19]. The overall rate of absorbed molecules would be:

(1) $$r_{absorb}\left(t\right)=Q\sum_{k=0}^{N_{K}}\frac{F_{hit}^{k}\left(t-\Delta t,t\right)}{min\left(t-t_{k},\Delta t\right)},$$

where *n* is the number of RX receptors, $R_{r}$ is the RX radius, $r_{s}$ is the receptor radius, *d* is the minimum distance between the TX and the RX, $N_{K}=\left\lfloor t/\Delta t\right\rfloor$ is the number of pulses transmitted up to *t*, and $F_{hit}^{k}\left(t-\Delta t,t\right)$ is the mean fraction of molecules arrived at the RX receptors in the interval $\left(t-\Delta t,t\right]$ and transmitted by the TX during the *k*-th pulse at time $t_{k}=k\Delta t$. According to the definition provided by Equation (14) in [15], we can rewrite this latter quantity as $F_{hit}^{k}\left(t-\Delta t,t\right)=F_{hit}^{r_{s},n}\left(max\left(0,t-t_{k}-\Delta t\right),t-t_{k}\right)$, where $F_{hit}^{r_{s},n}\left(t_{1},t_{2}\right)=F_{hit}^{r_{s},n}\left(t_{2}\right)-F_{hit}^{r_{s},n}\left(t_{1}\right)$, and $F_{hit}^{r_{s},n}\left(t\right)$, given by Equation (13) in [15] and reported below in (2), represents the fraction of molecules absorbed by the *n* receptors up to time *t* assuming a release of pulse at time t=0:

(2) $$\begin{array}{ccc} F_{hit}^{r_{s},n}\left(t\right) & = & \frac{R_{r}}{d}\frac{r_{s}n}{r_{s}n+\pi R_{r}}(1+\mathrm{erf}\left[\frac{R_{r}-d}{\sqrt{4Dt}}\right]-\;\exp[\left(d-R_{r}\right)\left(\frac{nr_{s}+\pi R_{r}}{\pi R_{r}^{2}}\right)+\\ & & Dt{\left(\frac{nr_{s}+\pi R_{r}}{\pi R_{r}^{2}}\right)}^{2}]\times \;\mathrm{erfc}\left[\frac{d-R_{r}+2Dt\left(\frac{nr_{s}+\pi R_{r}}{\pi R_{r}^{2}}\right)}{\sqrt{4Dt}}\right]) \end{array}$$

It is clear the existence of a complex and nonlinear dependency of the fraction of absorbed molecules in a time interval and the number of receptors *n* deployed on the RX surface.

We define as the arrival process to all receptors located on the RX surface the product of the rate of emitted molecules at the TX (Q/Δt) and the fraction of each burst that would be absorbed by an RX with absorbing receptors [15], and denote it as $\Lambda_{o}\left(t\right)$. In case of absorbing receptors, the rate of arrival is exactly the rate of absorption, that is, $\Lambda_{o}\left(t\right)=r_{absorb}\left(t\right)$, whereas the arrival rate per single receptor $\lambda_{o}\left(t\right)$ is simply this number scaled by *n*, as in (1):

(3) $$\lambda_{o}\left(t\right)=\frac{\Lambda_{o}\left(t\right)}{n}=\frac{r_{absorb}\left(t\right)}{n}=\frac{Q}{n}\sum_{k=0}^{N_{K}}\frac{F_{hit}^{k}\left(t-\Delta t,t\right)}{min\left(t-t_{k},\Delta t\right)}.$$

Taking into account the analysis carried out in [20], as already mentioned in the Introduction, we remove the simplifying assumption of having absorbing receptors and consider larger molecules. This means that we focus on reactive receivers [5], where the time for completing the reaction/internalization between the ligand and the receptor is usually non-negligible [6]. The consequence is that the resulting rate of absorption evaluated for the absorbing receiver in our model becomes the rate of *fresh, new* molecule arrivals from the TX. This means that we have two different contributions for computing the overall arrival rate:

- the arrivals of *new* molecules coming from the TX upon a new burst. This contribution is equal to $\lambda_{o}\left(t\right)$ in (1) for each receptor;
- the attempts of molecules that have found a receptor busy to make a again a bond with it, since after a bounce with a busy receptor, these ligands are still very close to it, given the movement dynamics into play. Clearly, this second contribution depends on the rate of *rejected*, i.e., not absorbed, molecules.

As we avoid making specific assumptions on the location of receptors on the RX surface, we just make the simplified assumption that all receptors are *virtually* located on the same point, as illustrated in Figure 1c.

In [9], we have already shown that, under a continuous flow of molecules, a Poisson process is a good model to approximate the arrival process of molecules at receptors. If we focus on a single receptor, the *new* arrivals can be modeled by a non-homogeneous Poisson process [9,11], with variable mean rate $\lambda_{o}\left(t\right)$, as in (3). Instead, the rate of rejected molecules, i.e., those that attempted to bind with a busy receptor, is denoted as $\lambda_{r}\left(t\right)$. A portion of these molecules escape ($\lambda_{ro}$) with probability $1-P_{c}$, whereas the others attempt again to bind with the receptor ($\lambda_{ri}$), where $P_{c}$ is defined as the capture probability. For modeling the *overall* arrival rate $\lambda_{o}^{\prime }\left(t\right)$ including both contributions, we use two approximations. First, given the values of quantities involved in the capture probability $P_{c}$, it results that

(4) $$P_{c}=F_{hit}^{r_{s},n,d^{*}}\left(\Delta t\right),$$

where the parameter $d^{*}$ in (4), which substitutes *d* in (2), does not represent anymore the distance between TX and RX center, but the distance of a particle to the RX center after bouncing over a busy receptor. The overall distance from the RX surface after the bounce will be obviously lower than the overall displacement of the particle during a simulation time step dt when it does not hit any obstacle. This implies that this value $d^{*}$ is dominated nearly always by

(5) $$d_{max}^{*}\approx \sqrt{3}\left(3\sqrt{2Ddt}\right)+R_{r},$$

where the term inside the brackets represents three times the standard deviation of a Brownian shift in a time step in a single direction, whereas $\sqrt{3}$ is due to the fact that the movement happens in 3D. Finally, the considered time interval is limited to Δt as it represents the time during which the effect of a pulse is more important. In our model, we set $d^{*}=d_{max}^{*}$. Differently from our previous work [14], we do not use the approximation of an absorbing receiver to model the probability of capture. In fact, although this approximation may be acceptable when the number of receptors *n* is quite large, in the order of thousands, it provides an overestimation when *n* decreases, especially for values well below 1000. Instead, as (2) and thus (4) also takes into account *n*; this approximation is much more reasonable. In Section 3, we analyze the impact of this approximation, showing that our model indeed matches simulation values quite well.

The second approximation is as follows. From elementary queuing theory, it is known that the overflow traffic does not follow a Poisson distribution, but a Pascal distribution, which exhibits a larger variance. Nevertheless, we approximate the overall molecule arrival rate per receptor as a non homogeneous Poisson process with rate

(6) $$\lambda_{o}^{\prime }\left(t\right)=\lambda_{o}\left(t\right)+\lambda_{ri}\left(t\right)\approx \lambda_{o}\left(t\right)\left(1+P_{c}\frac{\lambda_{o}\left(t\right)}{\mu +\lambda_{o}\left(t\right)}\right).$$

where the term $\frac{\lambda_{o}\left(t\right)}{\mu +\lambda_{o}\left(t\right)}$ represents the probability of finding a receptor busy due to incoming arrivals $\lambda_{o}\left(t\right)$. According to queuing theory, we define the per-receptor load as

(7) $$A_{o}\left(t\right)=\frac{\lambda_{o}^{\prime }\left(t\right)}{\mu }.$$

This implies that the per-receptor rejection rate $\lambda_{r}\left(t\right)$, according to queuing theory, is given by the product of the offered load and the rejection probability, that is,

(8) $$\lambda_{r}\left(t\right)=\lambda_{o}^{\prime }\left(t\right)\frac{A_{o}\left(t\right)}{1+A_{o}\left(t\right)}=\frac{A_{o}^{2}\left(t\right)}{1+A_{o}\left(t\right)}\mu ,$$

whereas the rate of absorbed molecules is given by

(9) $$\lambda_{a}\left(t\right)=\lambda_{o}^{\prime }\left(t\right)-\lambda_{r}\left(t\right)=\frac{\lambda_{o}^{\prime }\left(t\right)}{1+A_{o}\left(t\right)}=\frac{A_{o}\left(t\right)}{1+A_{o}\left(t\right)}\mu ,$$

When considering the whole RX, the aggregated average arrival rate is $\Lambda_{o}^{\prime }\left(t\right)=n\lambda_{o}^{\prime }\left(t\right)$, consequently the average aggregated rejection rate is equal to $\Lambda_{r}\left(t\right)=n\frac{A_{o}{\left(t\right)}^{2}}{1+A_{o}\left(t\right)}\mu$, and the average aggregate absorption rate is equal to $\Lambda_{a}\left(t\right)=n\frac{A_{o}\left(t\right)}{1+A_{o}\left(t\right)}\mu$. The cumulative number of rejections at RX can be evaluated as $N_{r}\left(t\right)=\int_{0}^{t}\Lambda_{r}\left(t\right)dt$, whereas the cumulative number of absorption as $N_{a}\left(t\right)=\int_{0}^{t}\Lambda_{a}\left(t\right)dt$. We will use these last two metrics—$Nr\left(t\right)$ and $N_{a}\left(t\right)$—to evaluate the goodness of the proposed model with respect to particle based simulations.

Finally, let us consider the expected performance in steady state conditions, that is, for $t\overset{}{\to }\infty$. In this case, it is easy to show that the rate of absorption of a RX with absorbing receptors would be equal to

(10) $$r_{absorb}=\frac{Q}{\Delta t}\frac{R_{r}}{d}\frac{r_{s}n}{r_{s}n+\pi R_{r}}.$$

As for our system, the steady-state expression for the absorption rate for the whole RX would be

(11) $$\Lambda_{a}=\frac{n\lambda_{o}^{\prime }}{1+A_{o}}.$$

In case the absorption time vanishes, that is, our system could be modeled as a RX with nearly absorbing receptors (i.e., $\mu \overset{}{\to }\infty$), it is easy to verify that the two models overlap, that is, $\Lambda_{a}\overset{\mu \overset{}{\to }\infty }{\to }r_{absorb}$, as $A_{o}\overset{\mu \overset{}{\to }\infty }{\to }0$.

To go further, it could be of interest to evaluate not only the average values, but also some other statistics. For instance, this may include the evaluation of the probability that at least a given fraction of receptors is busy in a specific time interval, in order to evaluate the effectiveness of drug delivery system. For this purpose, we follow the same approach proposed in [8]. In fact, at the nanomachine level, the number of receptors $n_{busy}\left(t\right)$ that established a bond with a ligand molecule can be modeled through a Binomial distribution $B\left(n,\pi_{r}\left(t\right)\right)$, as each cell has *n* receptors and each of them is occupied by a ligand molecules with the probability of having the receptor busy, that is, $\pi_{r}=\frac{A_{o}\left(t\right)}{1+A_{o}\left(t\right)}$, which coincides with the rejection probability for an M/M/1/1 queue. Obviously, the average number of busy receptors in the RX, $E\left[n_{busy}\left(t\right)\right]$, is simply equal to

(12) $$E\left[n_{busy}\left(t\right)\right]=n\times \pi_{r}\left(t\right)=\frac{nA_{o}\left(t\right)}{1+A_{o}\left(t\right)}.$$

However, as the evaluation of binomial distribution may become computationally demanding for large values of *n*, in that case it can be well matched by the normal distribution $N\left(n\pi_{r}\left(t\right),\sqrt{n\pi_{r}\left(t\right)\left(1-\pi_{r}\left(t\right)\right)}\right)$.

The proposed model can be applied to use cases in which it is necessary to model a flow of particles towards one or more targets, such as those relevant to drug delivery. For instance, by considering some specific drugs, such as competitive antagonist ones [21], i.e., those drugs that that compete with other ligands for a common binding site in a receptor to lock it, a complete drug response can be produced even with low receptor occupancy. Furthermore, additional receptors, not bound to drug molecules, are no longer needed to obtain this maximum response. Thus, in this case study it is possible to apply the model to evaluate the probability $P_{success}$ of having at least the minimum number of receptors $n_{\min}$ making bonds with drug molecules. Consequently, it is possible to evaluate the release rate able to achieve this target and thus the desired drug response. In this regard, when the number of receptors *n* is low, the most suitable model to describe the system response is the one using the Binomial distribution. In more detail, if the minimum number of receptors that should be occupied by drug molecules is $n_{\min}$, the success probability can be evaluated as

(13) Psuccessnmin=∑i=nminnniπri(1−πr)n−i.

From (13), it is possible to derive the optimal value of $\pi_{r}$ and, in turn, determine $A_{o}$ and thus, by using (7), $\lambda_{o}$. Once $\lambda_{o}$ is known, the release rate Q/Δt can be obtained from mathematical inversion of (3) (see also (10) and (11)).

Instead, if the number of receptors *n* is large, it is more computationally efficient to use the Gaussian approximation by resorting to the complementary error function to evaluate the success probability.

## 3. Numerical Results

The performance of the system has been evaluated by particle-based simulations carried out using the BiNS2 simulator [22], which is a Java package designed to simulate nano-scale biological communications in 3D. In particular, we have used the version which benefits from parallelized GPU acceleration, described in [13]. The simulation includes a TX and an RX in an unbounded diffusive environment. The main simulation parameters, together with their descriptions and values, are reported in Table 1. Instead, our proposed tool has been implemented in Matlab, without any special optimization. It uses the M/M/1/1 model to evaluate the performance (absorbed or rejected molecules) for a single receptor, leaving in postprocessing the procedure to aggregate results at the RX level. In more detail, we have built a simple simulator, which receives in input a Poisson process with mean rate realized by combining Equations (3) and (6). For each molecule arrival at the server (i.e., the receptor), we use the M/M/M/1/1 model to determine the outcome, as schematically illustrated in Figure 1. Finally, we count absorptions and rejections at receptor and RX level.

In order to compare the two approaches, we present the statistics relevant to absorptions and rejections at RX level evaluated by means of BiNS2 (labeled as *Sim*) and the proposed tool (labeled as *Model*).

**Figure 2:**
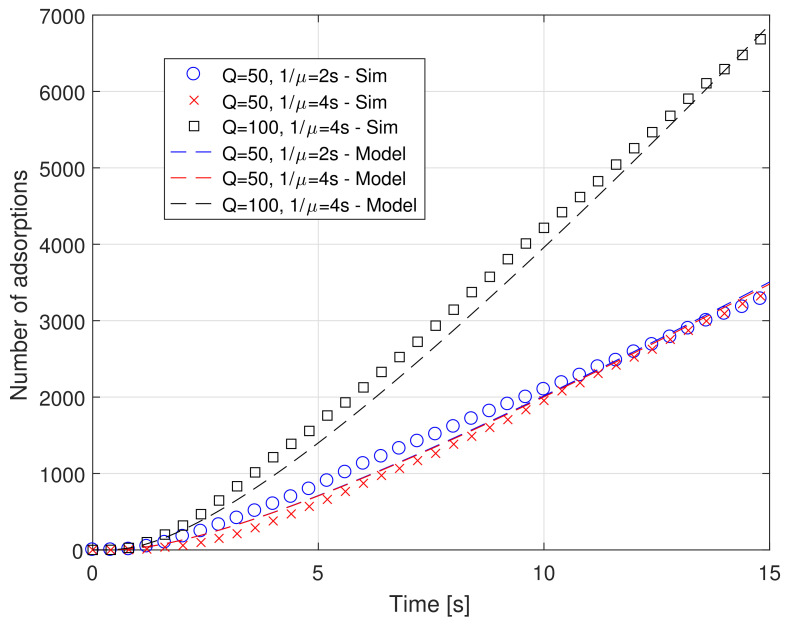
Absorbed molecules vs. time for different values of system parameters (burst size *Q* and absorption time 1/μ.

**Figure 3:**
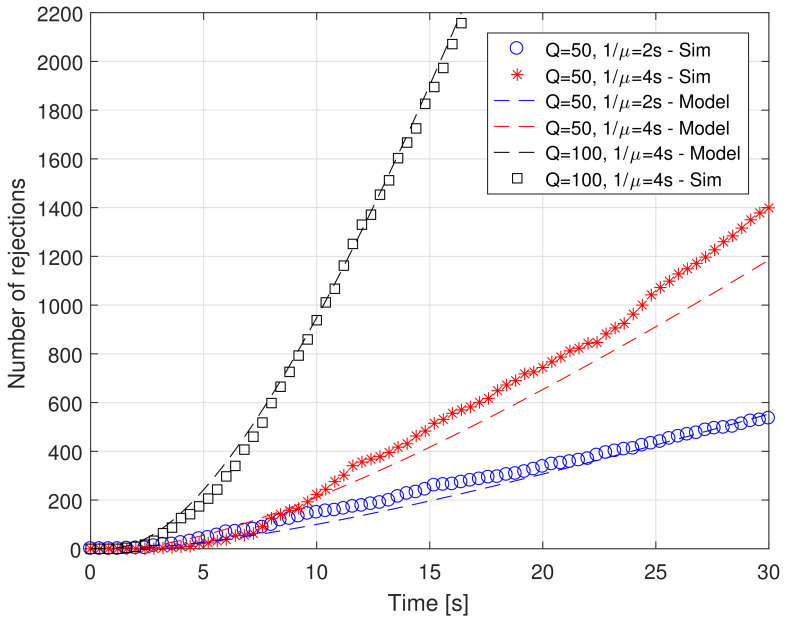
Rejected molecules vs. time for different values of system parameters (burst size *Q* and absorption time 1/μ.

Figure 2 shows the number of absorbed molecules as a function of time for both approaches, for different values of the trafficking times (2 and 4 s) and of the burst size *Q*, equal to 50 and 100 molecules emitted each 10 ms, which realizes a net emission rate equal to 5000 and 10,000 molecules/s, respectively. Instead, Figure 3 shows the number of rejected molecules as a function of time for both approaches, for different values of the parameters, as in Figure 2.

We can comment the two figures together, as they present very similar results. In particular, they show a very good agreement between the proposed model and simulation results in terms of both absorption and rejection rates for different values of system parameters *Q* and μ.

As for the simulated system, we can see that the impact of the trafficking time on the number of absorptions is almost negligible for *Q* equal to 50. This is true for both the proposed model and for the simulations. This means that the system is still not in saturation and any variation of the absorption time does not impact in a significant way. However, this is not completely true for rejections. In fact, an increase of the absorption time (1/μ) decreases the time during which the serving system, that is, the receptor, is available to form a new bond. Thus, increasing 1/μ implies increasing the number of rejections, as can be appreciated in Figure 3. When the emission rate increases, that is, *Q* becomes equal to 100, the effect on the number of absorptions becomes evident. In fact, increasing the release rate means increasing the concentration of ligands in the surrounding space and thus also close to the RX surface, and with a doubling of the arrival rate. The net effect is a doubling of the number of absorptions, meaning that the system is still not completely saturated. However, the number of rejections increases in a more significant way. For instance, considering the scenario with 1/μ=4 s, at the simulation time t=10 s the rejected molecules number ~200 for Q=50, whereas they jump to slightly less than 1000 for Q=100.

As additional comment applies. As both $\lambda_{a}\left(t\right)$ and $\lambda_{r}\left(t\right)$ are accurately approximated, it follows that the proposed model matches well also the overall arrival rate to the serving system, and thus the proposed approach is reliable in all its aspects.

As such an agreement holds not only in the steady state, but also in the transient phase (i.e., the first simulation seconds), the model can be used to reduce the simulation time in a number of complex use cases [23], where theoretical models are difficult to develop and particle-based simulations are possible but highly time-consuming and very demanding in terms of computing resources.

Thus, our simplified model can reproduce the aggregate behavior of thousands of receptors spread over the receiver surface, with a reduction in the simulation time in the order of $10^{4}$ times: from more than one day for particle-based simulations with BiNS2 to few seconds to simulate 1 s. In addition, we have to consider that particle-based simulation of such a system is increasingly demanding from the computation viewpoint, as the number of objects to manage (i.e., the released molecules in the simulation space) increases over time. For instance, after 10 s of simulated time, the number of releases molecules is $10^{6}$ when *Q* is equal to 100, whereas the amount of them being absorbed and thus removed from the simulation is lower than 5000. Instead, as the proposed system works with number values scaled by 1/n, simulation times are much more affordable. This is in short the reason why the simulation time significantly decreases: the simulator cannot handle all the released objects and their continuous movement, but just the molecules that *arrive* at receptors. This number depends not only on *Q* and Δt, but also on *n*. This means that the fewer the receptors able to bind to released ligands, the lower the simulation time when the proposed model is used.

We think it is useful to further investigate these concepts. We analyze the impact of the number of receptors *n* on the overall system model, from the arrival rate to the number of absorptions and its agreement with results obtained through particle-based simulations. For this purpose, we consider a number of receptors ranging in the interval from few tens (i.e., 50) to thousands (i.e., 10,000, the value used up to now and reported in Table 1). In order to make the performance figure comparable, we keep the generation rate at TX constant, that is, Q/Δt=50/0.01=5000 molecules per second.

**Figure 4:**
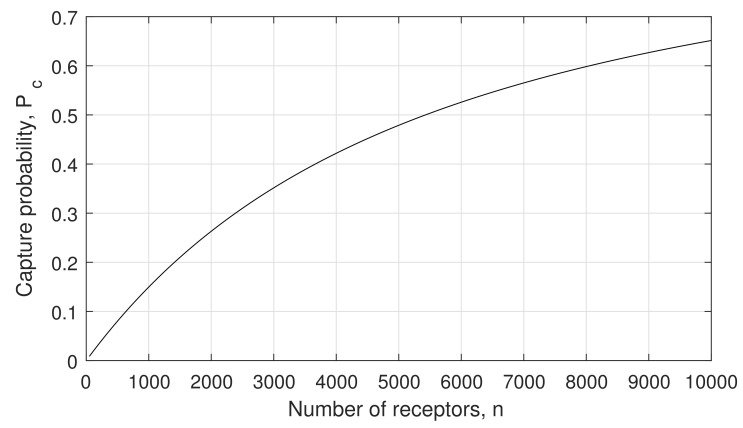
Capture probability $P_{c}$ as a function of the number of receptors, *n*.

We first investigate the effect of variable number of receptors on the capture probability $P_{c}$, as it has a direct impact on arrival rates $\Lambda_{o}^{\prime }\left(t\right)$ and $\lambda_{o}^{\prime }\left(t\right)$ through (6). Figure 4 shows the capture probability as a function of the number of receptors *n*. As expected, when their number is quite large, the capture probability is significant, reaching values in the order of 0.7 for n= 10,000. However, when n≤5000, then $P_{c}<0.5$, and for n≤1000, $P_{c}$ becomes well below 0.2. Finally, when the number of receptors is just few tens, the capture probability becomes negligible, taking values in the order to 1–2%. This is expected, as when the number of receptors becomes really low, a particle bouncing back on a busy receptor has a very small probability of finding another free receptor close to it. Its essential possibility of being absorbed consists of hitting the same receptor again and finding it available to form a bond.

**Figure 5:**
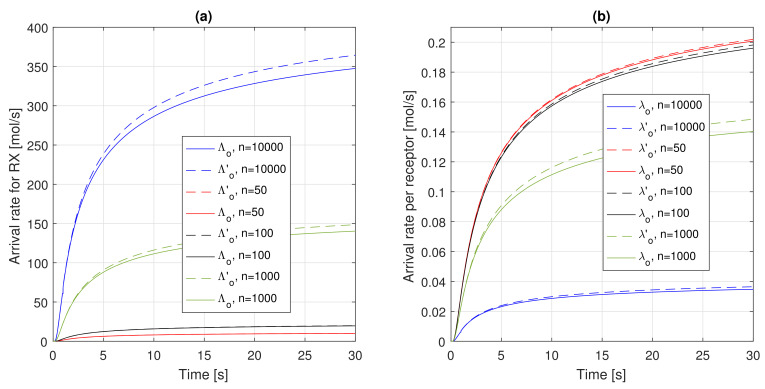
Arrival rates as a function of time for different values of the number of receptors, *n*: (**a**) $\Lambda_{o}^{\prime }\left(t\right)$ and $\Lambda_{o}\left(t\right)$, and (**b**) $\lambda_{o}^{\prime }\left(t\right)$ and $\lambda_{o}\left(t\right)$.

The net effect of this phenomenon is a reduction of the importance of the capture probability for low values of *n*, with $\Lambda_{o}^{\prime }\left(t\right)\overset{}{\to }\Lambda_{o}\left(t\right)$. This is confirmed by Figure 5. In more detail, Figure 5a presents aggregate values of arrival rates, whereas Figure 5b presents arrivals rates per receptor. From the analysis of the former, what was anticipated emerges: The impact of $P_{c}$ is significant for large values of *n* (5000 and 10,000 in the figure), and it becomes negligible for very low values of the number of receptors. In addition, a further comments is that the arrival rates, both $\Lambda_{o}^{\prime }\left(t\right)$ and $\Lambda_{o}\left(t\right)$, does not scale linearly with *n*, but follows a nonlinear pattern. In particular, when *n* is reduced by a factor 10 (i.e., from 10,000 to 1000, or from 1000 to 100), the corresponding aggregate arrival rate exhibits a much lower decrease. This is due to the strong nonlinearity of the function $F^{r_{s},n}\left(t\right)$, shown in (2). The consequence is that the arrival rate *per receptor*, that is, $\lambda_{o}^{\prime }\left(t\right)$ and $\lambda_{o}\left(t\right)$, exhibits an opposite behaviour, as they increase with decreasing *n*, as shown in Figure 5b. This can be explained by the fact that these models have their foundations in a receiver with absorbing receptors. In such a case, when the number of receptors decreases, the overall number of absorbed molecules clearly decreases. However, this means that an increasing concentration of molecules is present close to the RX surface, and thus the number of molecules absorbed by each receptor likely increases. As we use $F^{r_{s},n}\left(t\right)$ as the basis to model the particle arrivals, it is clear that we observe the same phenomenon, which is reasonable.

**Figure 6:**
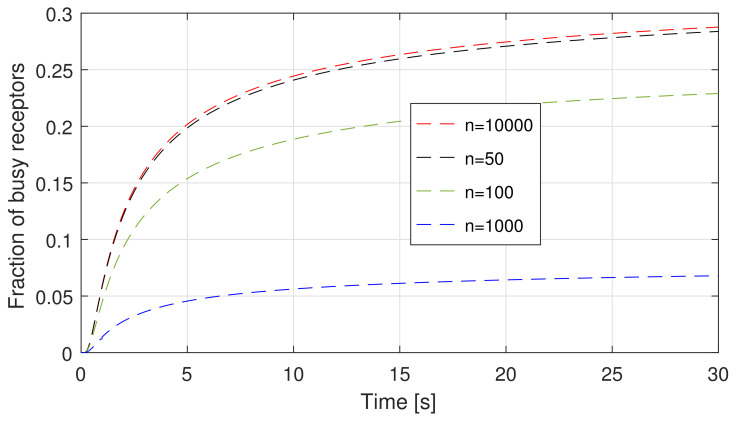
Average number of busy receptors as a function of time for different values of *n*.

In addition, a further comment is about the impact of the capture probability on $\lambda_{o}^{\prime }\left(t\right)$. In fact, for very low values of *n*, we have already mentioned that $P_{c}$ is negligible, thus we do not expect a significant difference between $\lambda_{o}^{\prime }\left(t\right)$ and $\lambda_{o}\left(t\right)$. This is confirmed by Figure 5b. Clearly, when n=1000, this values begins to be relevant for $\lambda_{o}^{\prime }\left(t\right)$, but for further increments, it follows an opposite behaviour. This is due to the fact that, even if $P_{c}$ takes large values (about 0.65 for n= 10,000) with a much larger number of receptors, the probability for a ligand to find a receptor not busy and thus available to form a bond is larger. Thus, the impact *per receptor* of $P_{c}$ starts becoming less important. This is also confirmed by a further metric, the average number of busy receptors ($\Lambda_{a}\left(t\right)/\mu$) as a function of time, which is plotted in Figure 6. In fact, it is evident that, by keeping the generation rate Q/Δt constant, if the system capacity increases (i.e., *n*), the load of the system, normalized to its capacity, decreases.

**Figure 7:**
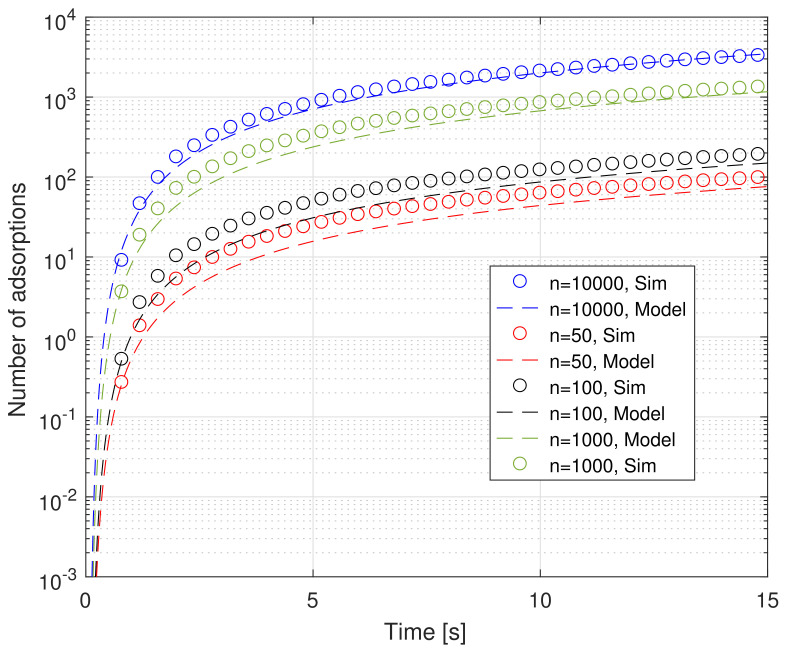
Average absorptions as function of time, with *n* as parameter, for both the proposed model and particle-based simulations.

Finally, Figure 7 shows the results needed for assessing the suitability of the model in matching particle-based simulations for very low values of the number of receptors *n*. It is quite evident that the proposed model is able to match the simulation results quite well. In order to make the comparison more evident, we used the logarithmic scale in the ordinate axis. Again, we can appreciate the fact that simulations are closely matched not only in the steady state, but also in the transient state. The small difference between the values obtained by using the model and those resulting from particle-based simulation is the cost of having a lightweight yet accurate model. It is based on some approximations to make it not only treatable, but also easy to simulate, instead of using time-consuming, full-fledged particle-based simulations.

## 4. Conclusions

In this paper, we have proposed a simplified model to evaluate the performance of local drug delivery systems in terms of number of absorbed molecules and saturation status of receptors on the receiver surface. The proposed model, based on the elementary M/M/1/1 queuing model for the single receptor, matches well with particle-based simulations obtained with a known simulation tool, the well-assessed BiNS2 simulation platform, providing a gaining in the execution time in the order of $10^{4}$ times.

Thus, the results of the proposed approach are promising and can be used to overcome difficulties in both modeling and simulation phase, guaranteeing a good level of reliability together with fast execution times. Alternatively, it can be effectively used also in more complex scenarios, where the system scale, in terms of number of receptors and/or amount of released particles, may require prohibitive computing times for both solving accurate theoretical model (e.g., due to explosion of the number of state space) or simulating the overall system.

## Figures and Tables

**Table 1:** Simulation parameters.

Symbol	Description	Value
$R_{r}$	Radius of TX/RX node 2.5 µm	
*n*	Amount of surface receptors (RX)	10,000
$r_{m}$	Radius emitted molecules	1.75 nm
*D*	Diffusion coefficient	$1.18\times 10^{-10}$ m${}^{2}$/s
$r_{s}$	Receptor radius (RX)	4 nm
1/μ	Trafficking time	2 or 4 s
Δt	Emission period (TX)	10 ms
$T_{S}$	Simulation time step	100 ns
*d*	RX-TX distance	26.5 µm
